# Remodeling of the Core Leads HIV-1 Preintegration Complex into the Nucleus of Human Lymphocytes

**DOI:** 10.1128/JVI.00135-20

**Published:** 2020-05-18

**Authors:** Guillermo Blanco-Rodriguez, Anastasia Gazi, Blandine Monel, Stella Frabetti, Viviana Scoca, Florian Mueller, Olivier Schwartz, Jacomine Krijnse-Locker, Pierre Charneau, Francesca Di Nunzio

**Affiliations:** a Department of Virology, VMV, Institut Pasteur, Paris, France; b Ecole Doctorale Frontiere de l’Innovation en Recherche et Education, CRI 75004, Université de Paris, Paris, France; c Unit for Service and Technology in Ultra-Structural Bio-Imaging, Center for Resources and Research in Technology, Pasteur Institute, Paris, France; d Paul Ehrlich Institute, Langen, Germany; e Department of Virology, UVI, Pasteur Institute, Paris, France; f Département Biologie Cellulaire et Infections, UIM, Pasteur Institute, Paris, France; g C3BI, USR 3756 IP CNRS, Paris, France; Ulm University Medical Center

**Keywords:** human immunodeficiency virus, integration, nuclear import/export, viral replication

## Abstract

Retroviral replication proceeds through obligate integration of the viral DNA into the host genome. In particular, for the HIV-1 genome to enter the nucleus, it must be led through the nuclear pore complex (NPC). During the HIV-1 cytoplasmic journey, the viral core acts as a shell to protect the viral genetic material from antiviral sensors and ensure an adequate environment for reverse transcription. However, the relatively narrow size of the nuclear pore channel requires that the HIV-1 core is reshaped into a structure that fits the pore. On the other hand, the organization of the viral CA proteins that remain associated with the preintegration complex (PIC) during and after nuclear translocation is still enigmatic. In this study, we analyzed the progressive organizational changes of viral CA proteins within the cytoplasm and the nucleus by immunogold labeling. Furthermore, we set up a novel technology, HIV-1 ANCHOR, which enables the specific detection of the retrotranscribed DNA by fluorescence microscopy, thereby offering the opportunity to uncover the architecture of the potential HIV-1 PIC. Thus, we combined the immunoelectron microscopy and ANCHOR technologies to reveal the presence of DNA- and CA-positive complexes by correlated light and electron microscopy (CLEM). During and after nuclear translocation, HIV-1 appears as a complex of viral DNA decorated by multiple viral CA proteins remodeled in a pearl necklace-like shape. Thus, we could describe how CA proteins are reshaped around the viral DNA to permit the entrance of the HIV-1 in the nucleus. This particular CA protein complex composed of the integrase and the retrotranscribed DNA leads the HIV-1 genome inside the host nucleus. Our findings contribute to the understanding of the early steps of HIV-1 infection and provide new insights into the organization of HIV-1 CA proteins during and after viral nuclear entry. Of note, we are now able to visualize the viral DNA in viral complexes, opening up new perspectives for future studies on virus’s fate in the cell nucleus.

**IMPORTANCE** How the reverse-transcribed genome reaches the host nucleus remains a main open question related to the infectious cycle of HIV-1. The HIV-1 core has a size of ∼100 nm, largely exceeding that of the NPC channel (∼39 nm). Thus, a rearrangement of the viral CA protein organization is required to achieve an effective nuclear translocation. The mechanism of this process remains undefined due to the lack of a technology capable of visualizing potential CA subcomplexes in association with the viral DNA in the nucleus of HIV-1-infected cells. By the means of state-of-the-art technologies (HIV-1 ANCHOR system combined with CLEM), our study shows that remodeled viral complexes retain multiple CA proteins but not an intact core or only a single CA monomer. These viral CA complexes associated with the retrotranscribed DNA can be observed inside the nucleus, and they represent a potential PIC. Thus, our study shed light on critical early steps characterizing HIV-1 infection, thereby revealing novel, therapeutically exploitable points of intervention. Furthermore, we developed and provided a powerful tool enabling direct, specific, and high-resolution visualization of intracellular and intranuclear HIV-1 subviral structures.

## Abstract (precis)

How the reverse-transcribed genome reaches the host nucleus remains a main open question related to the infectious cycle of HIV-1. The HIV-1 core has a size of ∼100 nm, largely exceeding that of the NPC channel (∼39 nm). Thus, a rearrangement of the viral CA protein organization is required to achieve an effective nuclear translocation. The mechanism of this process remains undefined due to the lack of a technology capable of visualizing potential CA subcomplexes in association with the viral DNA in the nucleus of HIV-1-infected cells. By the means of state-of-the-art technologies (HIV-1 ANCHOR system combined with CLEM), our study shows that remodeled viral complexes retain multiple CA proteins but not an intact core or only a single CA monomer. These viral CA complexes associated with the retrotranscribed DNA can be observed inside the nucleus, and they represent a potential PIC. Thus, our study shed light on critical early steps characterizing HIV-1 infection, thereby revealing novel, therapeutically exploitable points of intervention. Furthermore, we developed and provided a powerful tool enabling direct, specific, and high-resolution visualization of intracellular and intranuclear HIV-1 subviral structures.

## INTRODUCTION

Upon viral entry, the HIV-1 core is released into the host cell cytoplasm and starts its journey toward the nucleus (1, 2), helped or hampered by cellular factors (3–10). The viral core has a conical shape (120 nm by 60 nm by 40 nm), composed of ∼1,500 capsid (CA) monomers organized in hexamers or pentamers (11–13). This conical structure acts as a protective shell for the viral DNA (vDNA) against defense mechanisms of the host, such as nucleic acid sensors and nucleases (14–16), and can be the target of restriction factors, such as Trim5*α* and MX2 (9, 10). Furthermore, the viral CA seems to participate in two critical steps of the HIV-1 life cycle, nuclear translocation (4–7, 17) and integration (18–23). The viral core is considered fragile (24) and can exist only an intact or completely unbundled structure. However, a recent *in vitro* study highlighted the possibility that partial cores can be stabilized by host factors (25). Thus, HIV-1 CA can exist in different forms in infected cells: as intact cores, as monomers, pentamers, or hexamers, and probably as partial cores. Of note, the viral core largely exceeds the size (11–13) of the nuclear pore channel (26); thus, the core should disassemble before entering the nucleus. The immediate-uncoating model, which postulates a complete loss of the viral CA proteins (2), has been the most accredited model in the past, supported by the impossibility to copurify viral CA with other viral proteins at early time points due to its genetic fragility (24, 27). In contrast to this model, Dismuke and Aiken (20) were the first to propose a role for CA in post-nuclear entry steps, followed by other studies (21–23, 28). However, the viral CA protein was biochemically detected for the first time in the nucleus of macrophages and HeLa cells by Zhou and colleagues (23). The presence of CA in the nucleus was shown also by the analysis of individual time points of infection in fixed cells using surrogate viruses or by indirect viral labeling using live imaging (29–38); nonetheless, these studies failed to reveal the organization of CA during viral nuclear translocation. By demonstrating the importance of the CA to regulate and coordinate different steps of the HIV-1 life cycle, these findings prompted the development of small molecules (HIV-1 CA inhibitors), thereby promoting the development of a new class of antiretroviral drugs (39). It should be noted, however, that to date the presence of HIV-1 CA has never been shown in the nucleus of infected primary CD4^+^ T cells, the main target cells of HIV-1. In addition, the CA-positive structure associated with the reverse-transcribed DNA that enters the nucleus, in particular, in mitotic cells, remains enigmatic. The details of the morphology of intermediate states of viral replication complexes can be analyzed only at the nanometer level due to their small size (HIV-1 core, ∼100 nm) (11). Previous studies showed the possibility of visualizing HIV-1 cores in the cytoplasm by correlated light and electron microscopy (CLEM) under particular circumstances, such as in the presence of proteasome inhibition (40). Nevertheless, it has been impossible to visualize the intermediate states of viral replication complexes, as well as the organization of the viral preintegration complex (PIC) during a quasi-physiological infection at the nanometer level. The viral CA was shown to remain partially associated with the HIV-1 DNA after nuclear entry by confocal microscopy (30). Due to the subdiffraction size of CA-associated HIV-1 DNA, details of this complex could be revealed only by high-resolution imaging, until now unattainable due to the incompatibility of fluorescent DNA labeling techniques, such as DNA fluorescent *in situ* hybridization (FISH) or click chemistry (5-ethynyl-2′-deoxyuridine [EdU]), with transmission electron microscopy (TEM).

Our study provides new evidence on the organization of viral CA proteins before, during, and after nuclear translocation. To visualize intermediate states of viral replication complexes, we combined CLEM with a novel fluorescence approach, which we designated HIV-1 ANCHOR, to directly label HIV-1 DNA. The combination of both technologies, high-resolution electron and fluorescence microscopy, highlighted the configuration of the CA protein in viral complexes during the nuclear translocation step. We identified nuclear viral complexes, including the crucial components of a potentially functional PIC, the integrase (IN), and the retrotranscribed DNA.

We reveal multiple CA proteins in the nucleus of both HeLa cells and primary lymphocytes. We detected in the nuclear side of the NPC HIV-1 CA complexes that were organized in a pearl necklace-like shape, according to the distribution of gold-labeled CA, likely enabling the entry of the viral genome into the host nucleus, the cellular compartment of viral replication.

## RESULTS

### Viral RT correlates with HIV-1 CA and IN association.

**FIG 1:**
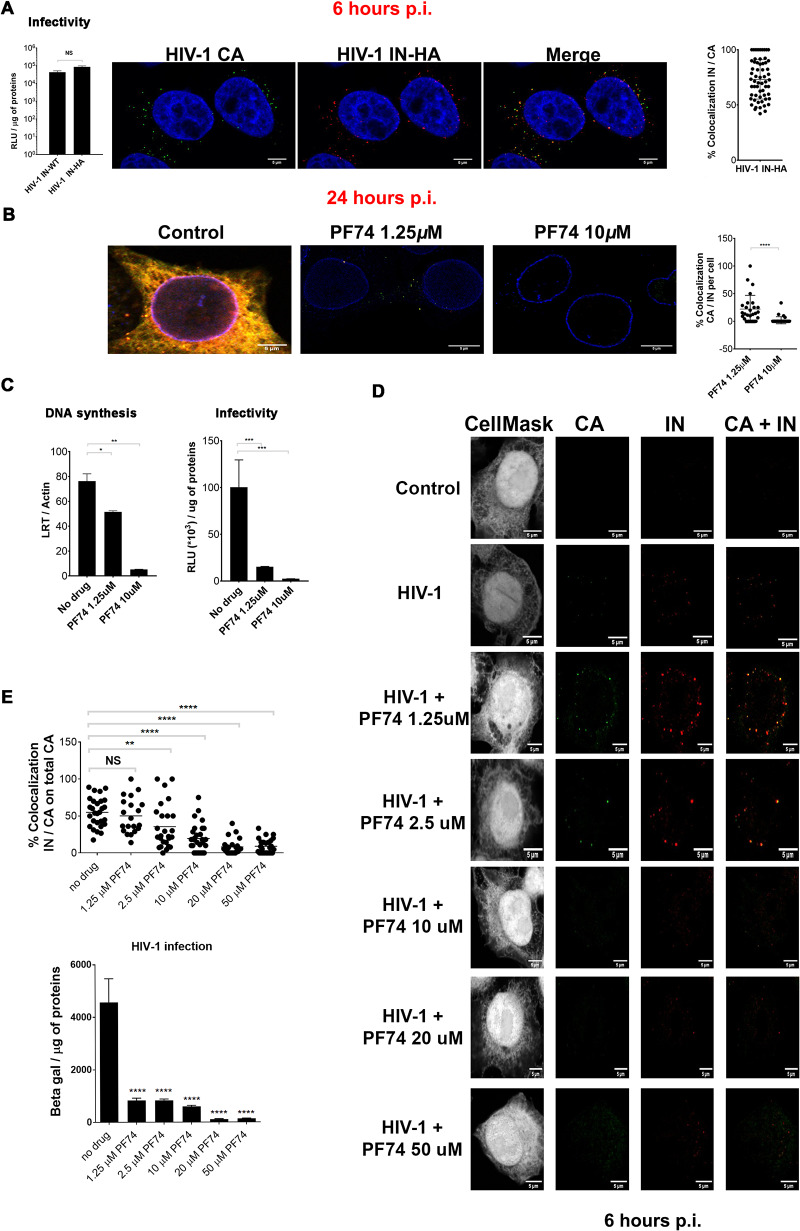
Viral reverse transcription correlates with HIV-1 CA and IN association. (A) Comparison of the infectivity of HIV-1 carrying the IN wild type or the IN fused to an HA tag, analyzed by beta-galactosidase assay, normalized by the amount of proteins. HeLa cells (10^6^ cells) were infected with 500 ng of p24 of HIV-1 ΔEnv IN_HA_/VSV-G, fixed at 6 h postinfection (p.i.), and labeled with antibodies anti-p24 (green) and anti-HA (red); host DNA is labeled by Hoechst (blue). Analysis of the percentage of IN/CA colocalization was analyzed by ImageJ and by Graph Pad Prism, version 7. (B) HeLa cells infected for 24 h in the presence or absence of the drug PF74 at a low dose (1.25 μM) or high dose (10 μM). Cells were fixed on 4% PFA and labeled with anti-p24, anti-HA, and anti-Nup153 antibodies. Colocalization between CA and IN was analyzed by ImageJ and by Graph Pad Prism, version 7. (C) DNA synthesis was evaluated by qPCR through the amplification of late reverse transcripts (LRT). The infectivity was analyzed at 48 h postinfection by beta-galactosidase assay and normalized by protein amount. (D) Effect of PF74 doses on HIV-1 CA and IN detections at 6 h postinfection in HeLa P4R5 cells by confocal microscopy. (E) Analysis of the percentage of IN/CA colocalization by ImageJ and by Graph Pad Prism, version 7 (graph on the top right). The level of infectivity has been evaluated at 48 h postinfection by beta-galactosidase assay, normalized for the quantity of proteins as reported in the histogram on the bottom right. Differences were analyzed by Graph Pad Prism, version 7 (Student’s *t* test), and considered statistically significant as follows: ****, *P* < 0.0001; ***, *P* < 0.001; **, *P* < 0.01; *, *P* < 0.1; NS, not significant. RLU, relative light units.

We analyzed the dynamics of the HIV-1 CA and IN association based on their colocalization and their link with viral retrotranscription. As the viral IN cannot be efficiently labeled using a direct antibody (Ab), we infected HeLa cells with HIV-1 containing a small hemagglutinin (HA) tag fused to the C terminus of the IN (HIV-1 ΔEnv IN_HA_/VSV-G, where VSV-G is vesicular stomatitis virus G protein) used to pseudotype viral particles (41). The genetically modified virus efficiently infected HeLa cells as well as primary CD4^+^ T lymphocytes similarly to the virus carrying the wild-type (WT) IN (Fig. 1A; see also Fig. 3B). This feature made this tagged virus a functional tool to study replication complexes in relation to the structure of the viral capsid. Such HA-labeled IN enabled us to study the association between CA and IN during viral infection using a quasi-WT virus. Cells fixed at 6 h postinfection showed ∼60 to 70% colocalization of viral CA with the viral IN (Fig. 1A and E). Next, we investigated the importance of the presence of CA and IN in cytoplasmic complexes within infected cells for reverse transcription by using a capsid-targeting small molecule, PF74. PF74 binds at the interface between the N-terminal domain (NTD) and the C-terminal domain (CTD) (19, 42) of CA, thus inhibiting HIV-1 infection by blocking nuclear entry in a reportedly bimodal dose-dependent mechanism (19, 42–44). It has been shown that reverse transcription (RT) can occur in the presence of a low dose of PF74 (<10 μM), while it was impeded at higher doses of this drug (42, 44). We challenged HeLa cells with HIV-1 in the presence or absence of PF74 using low (1.25 μM) and high (10 μM) doses. Then, we determined the number of IN/CA colocalization spots per cell at 24 h postinfection (Fig. 1B) by immunolabeling the viral CA and IN. When we applied a low dose of PF74, at 24 h postinfection we could detect more CA/IN colocalization (average of ∼20.5 colocalizations on a total of 595 spots detected) in comparison to the few CA/IN colocalizations observed in samples treated with a high dose of PF74 (average of ∼1.8 colocalizations on a total of 62.9 spots detected) (Fig. 1B). In agreement with other studies (19, 44, 45), a low dose of PF74 allowed reverse transcription to occur (∼60% with respect to the PF74-untreated infected sample) (Fig. 1C). However, since a low dose of PF74 interferes with the nuclear import of HIV-1, viral replication was not detected in these cells, as reported also by other investigators (Fig. 1C) (19, 44, 45). Instead, control cells (untreated with PF74) allowed active viral transcription (Fig. 1B and C). A high dose of PF74 caused a loss of the association between CA and IN, together with a loss of reverse transcription activity (Fig. 1B and C). We corroborated these results by carrying out dose-response experiments to check for the presence of CA/IN cytoplasmic complexes (at 6 h postinfection) and viral infectivity by beta-galactosidase assay (at 48 h postinfection) (Fig. 1D). These experiments showed a progressive loss of viral CA and viral IN association in a dose-response manner with respect to the PF74 drug. Our data indicate that CA/IN association correlates with viral reverse transcription (Fig. 1B to E).

### Remodeling of HIV-1 cores.

**FIG 2:**
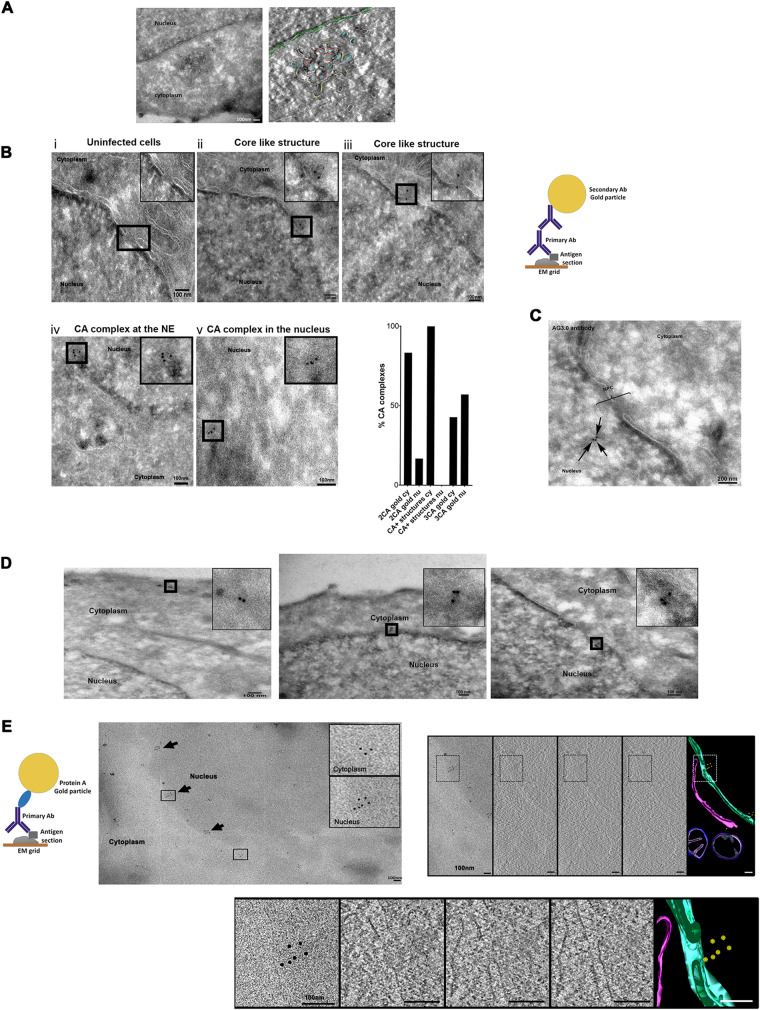
The road of viral CA complexes before and after nuclear translocation. (A) Images of HIV-1 virions englobed in the endosomes or cores escaping from the endosomes obtained by TEM. A 2D plane extracted from the tomogram of the endosomes containing viral particles is shown on the left. In the right-hand image, the cell volume was reconstructed (red, envelope; blue, cores; yellow, borders of the endosome; green, nuclear membrane). (B) Examples of structures resembling core-like structures docked at the NPC by TEM. A negative control based on uninfected cells is shown in frame i. Frames ii and iii show core-like structures docked at the NPC detected by CA labeled with two gold 10-nm particles revealing the antibodies anti-p24 (NIH183-H12-5C) attached. Frames iv and v show intranuclear CA complexes (scale bar, 100 nm). The schema at the right shows the labeling method, with the primary Ab anti-CA and secondary Ab conjugated to gold particles. Quantification analysis of the gold particle-labeled CA distribution in viral complexes (∼42) located in the cytoplasm (cy) or in the nucleus (nu) was performed. The percentage is shown of CA complexes detected by CA labeled with two gold particles (2CA) associated or not to a core-like shape and CA labeled with three gold particles (3CA) detected in the cytoplasm or in the nucleus. (C) HeLa P4R5 cells infected with HIV-1 IN_HA_/VSV-G. The sections were prepared and immunolabeled as described for panel B, using a primary antibody anti-CA (AG3.0). Black arrows indicate gold particles of 10 nm conjugated to a secondary Ab. Scale bar, 200 nm. (D) HeLa P4R5 cells infected with HIV-1 IN_HA_ carrying the WT envelope. The sections were prepared and immunolabeled as described for panel B. The areas containing the viral CA complexes are enlarged in the boxed areas. Scale bar, 100 nm. (E) Schema of the labeling method, with the primary Ab anti-CA and protein A-gold. Cryo-electron microscopy and immunogold labeling were used. The image shows several CA complexes (black arrows), each one with multiple CA proteins revealed by gold particles. The magnified views in the boxed areas display the differences in the gold distribution between outside and inside the nucleus. Images were obtained using an antibody against HIV-1 CA, followed by incubation with protein A coupled to 10-nm gold particles. A tomogram of a representative intranuclear CA complex, highlighted in a black rectangle, is shown. Sections were imaged in a T12 FEI electron microscope with tomography capabilities. The tomogram of the cell volume was reconstructed and manually segmented using IMOD. The upper panels, showing a larger view of the cell, starting from the left contain several planes (numbers 31, 43, and 49 out of 91 total slices) of the tomogram and the segmentation obtained from the reconstructed tomographic cell volume containing the gold-labeled CA complexes (yellow), nuclear envelope (green), endoplasmic reticulum (magenta), and mitochondria (purple). The bottom row shows magnified views of the boxed areas shown on the upper panels.

Next, we sought to visualize in more detail the cytoplasmic structures positive for both CA and IN. We infected HeLa P4R5 cells with 500 ng of p24 per million of cells. The asynchronous HIV-1 infection and the specific time of fixation (6 h postinfection) allowed visualization of different states of viral replication complexes by electron microscopy (EM). We observed several seemingly intact virions residing in endosomes (Fig. 2A), consistent with the entry pathway engaged by HIV-1 pseudotyped with VSV-G envelope (46). The intact particles in endosomes contained conical cores, while membrane-free cores, seemingly released from these organelles, were detected in the cytoplasm (Fig. 2A). However, to demonstrate the detection of free cores in the cytoplasm, sections were labeled with anti-CA and a secondary antibody coupled to 10-nm gold particles (Fig. 2B). CA-positive viral structures were usually decorated with two gold particles (Fig. 2B, frames ii and iii), although the viral core was composed of multiple CA monomers. Uninfected cells labeled with anti-CA and a secondary antibody labeled with gold particles revealed only background labeling, showing the specificity of the antibody (Fig. 2B, frame i). During and after the nuclear viral entry, conical core-like structures were not detected, and instead the immunolabeling decorated a different CA-positive pattern (Fig. 2B). On the cytoplasmic side of the nuclear pore, we could detect irregular shapes similar to core-like structures decorated with anti-CA (Fig. 2B, frames ii and iii). In the nucleus, however, the same antibody outlined pearl necklace-like shapes, composed of multiple CA proteins (Fig. 2B, frames iv and v). Cytoplasmic viral complexes featured CA typically decorated by two gold particles (∼80%) whereas the nuclear complexes were labeled on average by three particles (Fig. 2B). The detection of these particular nuclear CA forms was not antibody dependent because similar results were obtained with an alternative antibody against CA (AG3.0) (Fig. 2C). Importantly, the difference in gold distribution was also seen with wild-type HIV-1 (Fig. 2D), implying that it did not depend on the route of viral entry. By cryo-EM, we confirmed the presence of multiple CA complexes labeled with gold particles, forming a pearl necklace-like shape inside the nucleus of infected cells (Fig. 2E). These elongated CA complexes are better resolved by cryo-EM (47) and detected by more gold particles when protein A coupled to gold was used instead of a secondary antibody (Fig. 2E). This likely related to a better affinity of the protein A for primary antibodies than secondary gold-conjugated antibodies. Importantly, different labeling methods highlighted similar structures in the nucleus and confirmed the increase in gold labeling of CA in viral complexes after nuclear entry (Fig. 2B to E).

Taken together, our data indicate that the viral core undergoes a morphological rearrangement during nuclear entry coinciding with increased CA labeling, based on EM, likely due to increased accessibility of the antibody.

### Pearl necklace-like complexes contain IN and are present in the nucleus of CD4^+^ T cells.

**FIG 3:**
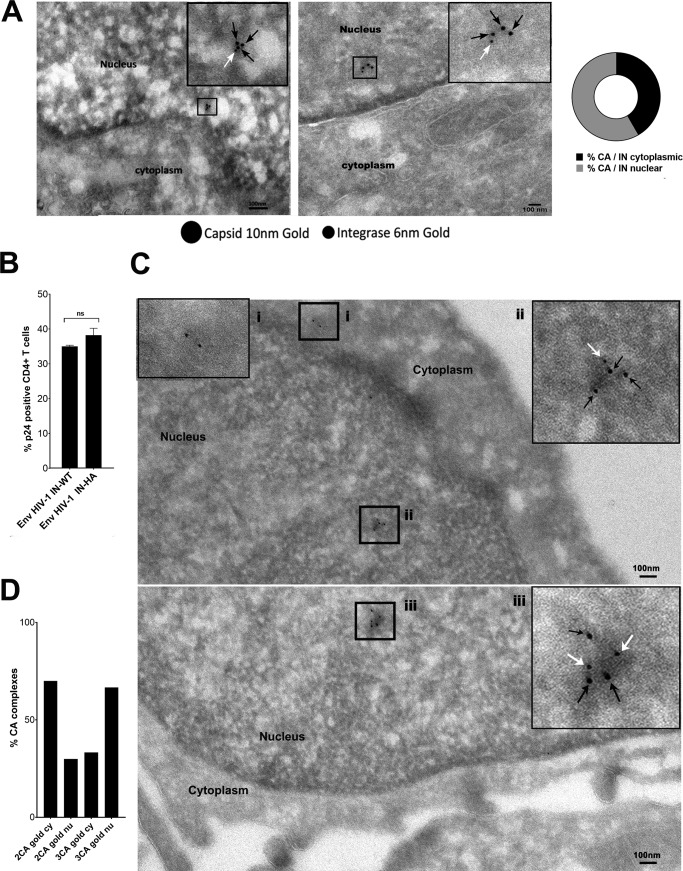
CA protein multimers are associated with IN inside the host nucleus. (A) Some CA complexes detected at 6 h postinfection contain IN. The double labeling of gold against primary antibodies anti-p24 (10 nm) and anti-HA (to label IN) (6 nm) highlights the association of both proteins particularly inside the nucleus of HeLa cells. CA/IN association in the cytoplasm (∼41%) and in the nucleus (∼58%) was calculated and is represented in the graph on the right. (B) Primary CD4^+^ T cells were isolated from healthy donors and infected with 4,000 ng of p24 of HIV-1. Infectivity was compared between primary CD4^+^ T cells (calculated as the percentage of positive p24 cells analyzed by cytofluorimetry) and wild-type enveloped viruses carrying the wild-type IN or the IN fused to an HA tag. ns, not significant. (C) CD4^+^ T cells were challenged with HIV-1 IN_HA_/VSV-G, and samples were processed for immunoelectron microscopy after 9 h from infection and stained with antibodies against capsid and HA for the integrase. Magnified insets represent CA complexes labeled with two gold particles located in the cytoplasmic area (i) and CA complexes labeled with three gold particles and containing integrase as well (ii and iii). The black arrows indicate capsid labeled with 10-nm gold; the white arrows point to the integrase labeled with 6-nm gold particles. (D) Analysis of the percentage of CA proteins labeled with gold cytoplasm (cy) and the nucleus (nu) containing CA labeled with two (2CA) or three (3CA) gold particles, respectively.

The presence of CA gold-labeled complexes in the nucleus of HeLa cells suggests that they are part of the viral complexes formed during nuclear translocation. Thus, to further characterize the composition and the spatial distribution of HIV-1 complexes inside and outside the nucleus, we labeled CA and IN with different sizes of colloidal gold conjugates (6 nm and 10 nm, respectively) and imaged them by TEM (Fig. 3A). IN was more frequently associated with intranuclear CA structures (Fig. 3A) that were detected by CA labeled with three or more gold particles than by CA labeled with two gold particles. To investigate if these complexes could be observed also in natural HIV-1 target cells, we repeated the experiments in primary CD4^+^ T cells, the natural HIV-1 host cells (Fig. 3B). We observed viral complexes labeled by two gold particles in the cytoplasm of CD4^+^ T cells derived from healthy donors at 9 h postinfection (Fig. 3C, frame i). The nuclear CA complexes detected by multiple gold particles were also observed in these cells (Fig. 3C, frames ii and iii). Furthermore, the difference between the cytoplasmic and nuclear CA gold labeling patterns was similar in HeLa cells and in primary lymphocytes (Fig. 2B and 3D).

Our results show that viral CA complexes associated with viral IN can be found in the nuclei of HeLa cells and also in those of natural HIV-1 target cells, i.e., primary lymphocytes. Thus, the formation of the CA nuclear complexes was not cell dependent, implying that they represent an important feature for the nuclear journey of HIV-1.

### Specific detection of retrotranscribed DNA in infected cells by the HIV-1 ANCHOR system.

**FIG 4:**
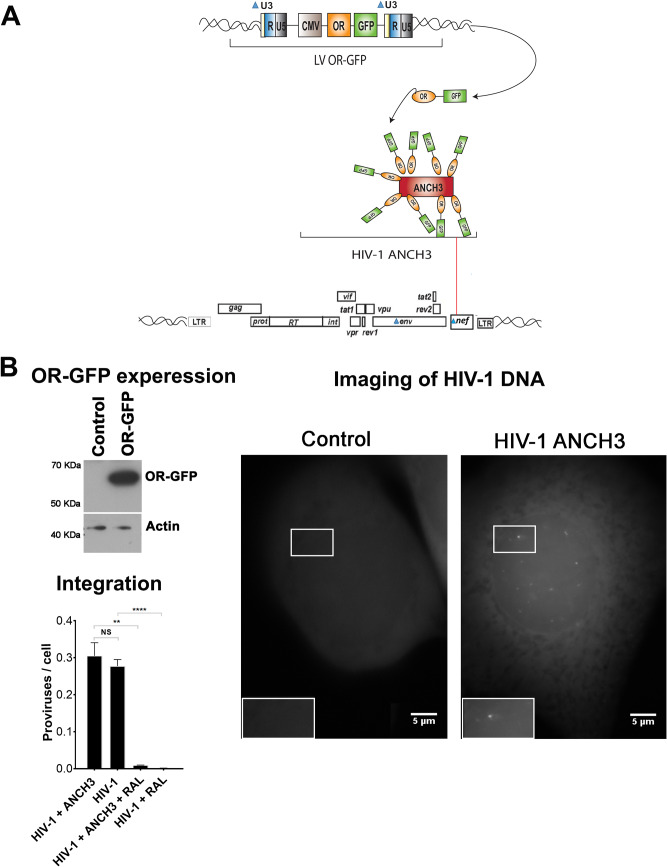
Detection of the retrotranscribed HIV-1 DNA in infected cells. (A) Schema of the HIV-1 ANCHOR system based on lentiviral vectors carrying on the OR-GFP cDNA under the control of CMV promoter (LV OR-GFP) and HIV-1 containing the ANCH3, the target sequence of OR proteins (HIV-1 ΔEnv IN_HA_ ΔNef ANCH3/VSV-G). (B) HeLa P4R5 cells were transduced with LV OR-GFP. The efﬁciency of OR-GFP expression was monitored by Western blotting using antibody against GFP. As a loading control, samples were also blotted using antibody against actin. HeLa cells stably expressing OR-GFP infected or not with HIV-1 ΔEnv IN_HA_ ΔNef ANCH3/VSV-G (MOI of 50) were imaged by fluorescence microscopy at 24 h postinfection using a water immersion objective in epifluorescence. The number of proviruses was detected by Alu-PCR on HeLa P4R5 OR-GFP cells infected with HIV-1 or HIV-1 ANCH3.

**FIG 5:**
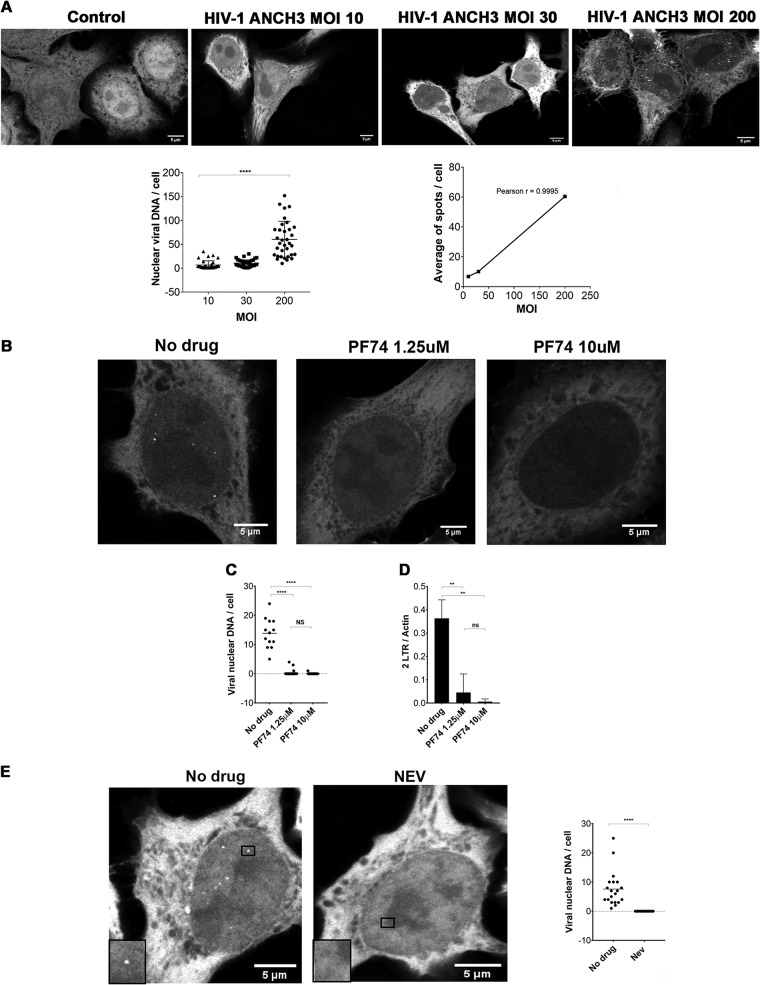
Specificity of HIV-1 ANCHOR system to detect the retrotranscribed DNA. (A) HeLa P4R5 cells stably transduced with LV OR-GFP were infected at different MOIs of HIV-1 ANCH3 and imaged after 24 h by confocal microscopy. The number of nuclear viral DNA spots per single GFP^+^ cell was analyzed in 2D by ImageJ. Correlation analysis and the Pearson’s coefficient as well as statistical analysis were performed by Graph Pad Prism, version 7 (analysis of variance test). (B) HeLa P4R5 cells infected at an MOI of 50 with HIV-1 ΔEnv IN_HA_ ΔNef ANCH3/VSV-G in the presence or absence of PF74 (low dose, 1.25 μM; high dose, 10 μM). Cells were imaged by confocal microscopy at 24 h postinfection. (C) Individual spots inside the nuclei were manually counted and statistically analyzed in 2D by Graph Pad Prism, version 7. Statistical significance was calculated using a two-tailed Student's *t* test (****, *P* < 0.0001; NS, nonsignificant). (D) Viral nuclear import was evaluated by qPCR (2LTRs) and normalized by actin. Statistical analysis was calculated by Graph Pad Prism, version 7, using a two-tailed Student's *t* test (****, *P* < 0.0001; **, *P*< 0.01; ns, not significant). (E) Confocal microscopy of intranuclear spot detection in HeLa P4R5 OR-GFP challenged at an MOI of 30 with HIV-1 ANCH3 in the presence or absence of NEV at 24 h postinfection. 2D statistical analysis of a manual count of intranuclear spots was performed by Graph Pad Prism, version 7. All data are representative of two or more independent experiments.

To investigate if the observed CA structures, detected during and after nuclear translocation, represent potential PICs, we analyzed whether the retrotranscribed viral genome was present in these complexes. The rarity of viral events during nuclear import made their visualization particularly difficult. Labeling of the retrotranscribed viral DNA has been a major challenge, and only partial success has been achieved using DNA FISH, a ViewHIV (direct visualization) assay, multiplex immunofluorescent cell-based detection of DNA, RNA, and protein (MICDDRP), or EdU in fixed cells (18, 29–31, 48–51). These DNA fluorescent labeling methods are all incompatible with the TEM technique. This limitation impeded the characterization of the PIC during nuclear translocation. To overcome this drawback, we set up a new system that allowed direct tracking of viral retrotranscribed DNA in CA-positive complexes. We adapted ANCHOR technology (NeoVirTech [NVT]) (52, 53) to visualize HIV-1 DNA (Fig. 4A). The ANCHOR technology consists of a bipartite system derived from a bacterial *parABS* chromosome segregation machinery. This is composed of a target DNA sequence, ANCH3, which is specifically recognized by the OR protein, a modified version of the bacterial *parB* protein (54, 55), fused to green fluorescent protein (GFP). We cloned ANCH3 sequence in the HIV-1 genome (HIV-1 ANCH3) to ensure direct labeling of the retrotranscribed viral DNA in a highly specific manner (the human genome of the host cells lacks the ANCH3 sequences). We used this virus to infect HeLa cells previously transduced with a lentivirus (LV) carrying OR-GFP cDNA (Fig. 4A and B). Our results by fluorescence microscopy revealed that HIV-1 ANCH3 was recognized by OR-GFP fusion proteins that accumulated on the target sequence, resulting in the formation of a bright fluorescent spot (Fig. 4B). OR-GFP has no nuclear localization sequence (NLS) and therefore freely diffuses in the cell, with predominant localization in the cytoplasm (53). Upon infection, OR-GFP was efficiently visualized in complex with the retrotranscribed viral DNA, particularly in the nucleus (Fig. 4B). Of note, cells infected with HIV-1 ANCH3 or with the untagged HIV-1 showed similar numbers of proviruses by Alu-PCR; thus, the two viruses behaved similarly during integration or the steps of infection prior to integration (Fig. 4B). Importantly, HIV-1 ANCHOR permitted tracking of HIV-1 DNA by live-cell imaging. Hence, we could follow the fate of the viral DNA from the reverse transcription step onward. Indeed, this technology enabled us to follow the HIV-1 DNA for more than 70 h in living cells by epifluorescence (see Movies S1A and S1B in the supplemental material) or by three-dimensional (3D) or two-dimensional (2D) spinning-disk microscopy (Movies S2A to S2C). Via live-imaging technologies we imaged infected cells to obtain complete information about the full cellular volumes. Next, to pinpoint the specificity of the HIV-1 ANCHOR system to detect exclusively HIV-1 DNA, we infected HeLa OR-GFP cells at different multiplicities of infection (MOIs) with HIV-1 ANCH3. We observed a linear correlation between MOI and the number of nuclear vDNA spots in GFP-positive (GFP^+^) infected cells (Pearson’s coefficient of ∼1) (Fig. 5A). The total numbers of intranuclear spots analyzed for each condition were 2,054 for 34 GFP^+^ infected cells at an MOI of 200, 393 for 38 GFP^+^ cells at an MOI of 30, and 290 for 44 GFP^+^ cells at an MOI of 10. Averages of nuclear spot counts were calculated for single conditions (average for an MOI of 10, 6.7 spots; for an MOI of 30, 10.07 spots; for an MOI of 200, average of 60.4 spots) (Fig. 5A). In addition, we infected cells in the presence of drugs, PF74 or nevirapine (NEV; inhibitor of RT). First, we challenged HeLa cells expressing OR-GFP with HIV-1 ANCH3 for 24 h without drug or in the presence of low and high doses of PF74 (Fig. 5B and C). Both doses of PF74 blocked viral nuclear entry (Fig. 5D) (44). We detected the viral DNA inside the nucleus mainly in the absence of PF74 (Fig. 5B and C; Movies S3A to S3C), in agreement with nuclear import data obtained by quantitative PCR (qPCR) (Fig. 5D). Total intranuclear spot counts were analyzed for each condition (no drugs, 180 spots in 13 GFP^+^ cells; PF74 low dose, 8 spots in 28 GFP^+^ cells; PF74 high dose, 1 spot in 27 GFP^+^ cells) (Fig. 5C). These results were confirmed also when the nevirapine was used. We counted intranuclear spots in 20 cells per condition, and we obtained the following results: 152 nuclear spots in the absence of NEV against 0 detections in the presence of the drug. Thus, nuclear punctae containing HIV-1 DNA were found only in NEV-untreated cells (Fig. 5E; Movies S4A and S4B). Overall, these observations demonstrated that HIV-1 ANCHOR technology faithfully tracked the retrotranscribed viral DNA.

### HIV-1 CA decorates the retrotranscribed viral DNA during nuclear translocation.

**FIG 6:**
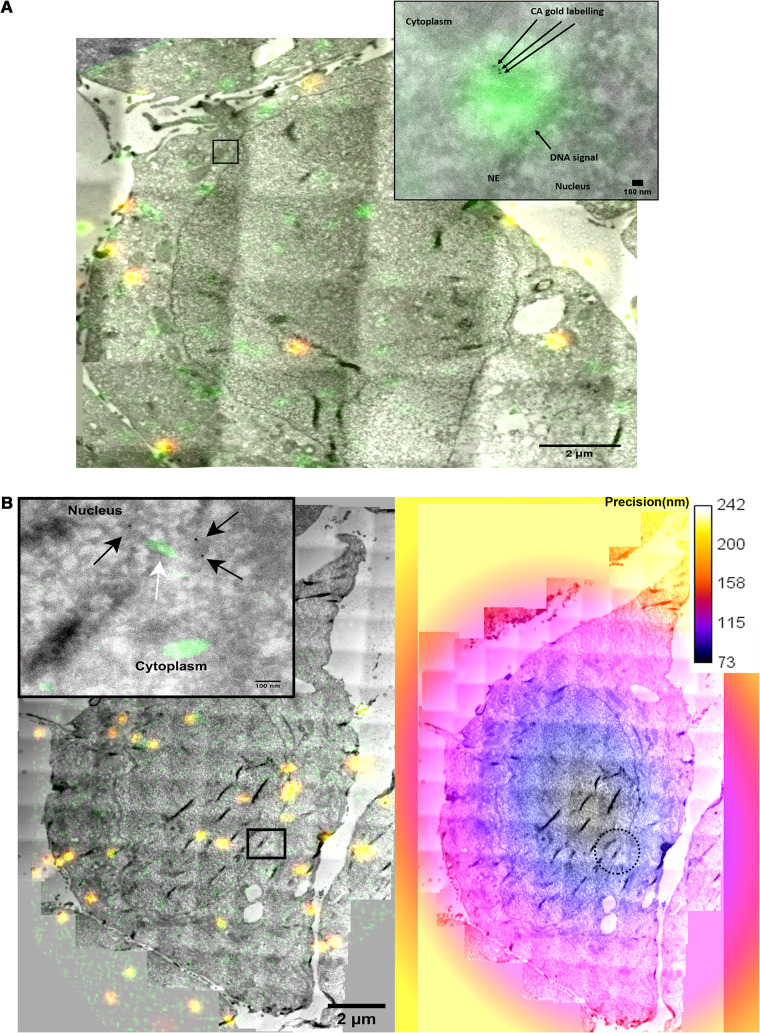
Multiple CA proteins leading retrotranscribed DNA detected by CLEM. (A) HeLa P4R5 cells transduced with OR-GFP were infected with HIV-1 ANCH3 and processed for correlative light and electron microscopy (CLEM). To achieve a precision correlation, fluorescent beads of 200 nm were added, visible also by EM. CLEM results on HeLa P4R5 OR-GFP cells at 6 h postinfection show the GFP signal (green), revealing the location of HIV-1 DNA. The signal of OR-GFP was amplified by using an antibody against GFP. HIV-1 CA was detected by the primary antibody anti-CA and a secondary gold-conjugated antibody (10-nm gold particles). The yellow signals correspond to the beads emitting in the green and in red channels. On the top right, a magnified view of the boxed area is shown. The green signal indicates the location of viral DNA, associated with the gold-labeled CA (dark dots). (B) Another biological replicate of CLEM shows multimers of CA proteins (dark dots) shrouding the vDNA (green signal) during nuclear translocation (left). The panel on the right shows an example of the precision correlation process applied. The precision of the correlation between TEM and fluorescence images was estimated with the ecCLEM plug-in in the Icy software environment. The calibration bar represents the precision achieved (in nanometers) by the different areas of the cells. The dashed circle shows the area enlarged in the boxed area of the panel on the left.

**FIG 7:**
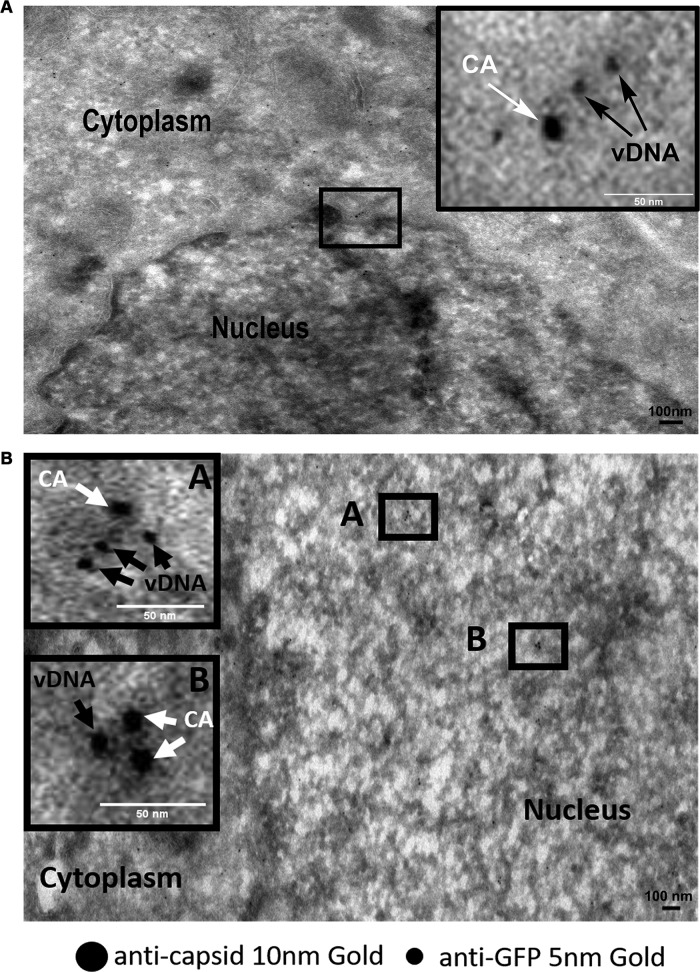
Viral complexes composed of CA and retrotranscribed DNA near the NE and inside the nucleus. (A) Double gold labeling coupled with TEM shows CA/OR-GFP (viral DNA) as part of the same complex near the NE. Viral DNA is detected by the presence of clusters formed by multiple OR-GFP proteins bound to ANCH3 sequence cloned in HIV-1 genome. OR-GFP proteins are labeled by the same primary antibody against GFP used in CLEM and a secondary antibody conjugated with 5-nm gold particles. HIV-1 CA is revealed by a primary antibody against CA (NIH183-H12-5C) and a secondary antibody conjugated with gold (10-nm particles). Scale bar, 100 nm. (B) Intranuclear viral complexes contain CA and viral DNA detected by double gold labeling coupled with TEM. Scale bar, 100 nm.

Our ultimate goal was to characterize the CA- and vDNA-positive complexes during and after nuclear translocation. One of the advantages of the OR-GFP system is its compatibility with TEM. Since this system is based on the interaction of OR protein with the ANCH3 sequence, we were able to apply the approach of correlative light and electron microscopy. Thus, we coupled HIV-1 ANCHOR to immunogold labeling to investigate if the viral DNA was part of the pearl necklace-like CA shapes previously detected by TEM (Fig. 2B, frames iv and v, Fig. 2C, D, and E, and Fig. 3A and C, frames ii and iii). We transduced HeLa cells with LV OR-GFP, and 3 days later we challenged these cells with HIV-1 ANCH3 for 6 h. Cells were fixed and prepared for EM-immunolabeling to test if structures positive for CA also contained viral DNA. Hence, we observed HIV-1 CA complexes near the nuclear pore complex (NPC) at 6 h postinfection. These CA complexes were positive for DNA, as observed by the correlative method (Fig. 6A). CA-positive complexes typically featuring CA decorated by three gold particles were also associated with HIV-1 DNA during nuclear translocation (Fig. 6B). The error of correlation between the two images (EM and fluorescence) has been calculated by the ecCLEM plug-in of Icy software (56, 57) to be theoretically ∼70 nm. CLEM results show a more elongated CA-positive form associated with the viral DNA during nuclear translocation (Fig. 6B) than the CA- and DNA-positive complex observed in the cytoplasm near the nuclear envelope (NE) (Fig. 6A). These results imply that this form of a viral complex can fit the size of the NPC and translocate through the pore to reach the host nuclear environment. Additionally, we performed a dual-gold-labeling experiment to detect viral complexes in the nucleus. We used different sizes of gold particles to label the viral DNA through OR-GFP (anti-GFP, 5-nm gold) and the viral CA (10-nm gold). Interestingly, we detected multiple gold particles labeling the viral DNA (5-nm particles) associated with CA (10-nm particles) adopting a linear configuration at the NE (Fig. 7A). This morphology corroborated the form of the PIC detected by CLEM (Fig. 6B). We were also able to reveal complexes formed by the viral DNA associated with HIV-1 CA in the nucleus of infected dividing cells (Fig. 7B). These data are in line with our CLEM results showing that viral complexes containing the retrotranscribed DNA can retain several CA proteins even after nuclear translocation.

Overall, results obtained by TEM and by CLEM highlighted the shape of a potential HIV-1 PIC during and after the nuclear entry step. Importantly, the detected viral complexes contain all required components for the integration, such as the integrase, DNA and, surprisingly, multiple CA proteins.

## DISCUSSION

The presence of viral proteins as well as the detection of double-stranded DNA (dsDNA), during nuclear translocation, strongly supports the notion that the HIV-1 core must undergo structural changes to be able to cross the NPC as the pore is too narrow to allow its passage as an intact core. Thus, the organization of viral CA complexes during the early steps of HIV-1 infection is still unknown due to the lack of appropriate tools to study this event. Our study provides some new evidence on the viral remodeling of HIV-1 core occurring prior to, during, and after viral nuclear entry. Importantly, we confirmed such observations in the main physiological target cell of HIV-1, CD4^+^ T cells. We were able to quantify a different distribution of CA gold labeling by comparing cytoplasmic and nuclear structures (Fig. 2B), supporting a viral CA remodeling process during nuclear entry. We observed that multilobe structures resembling core-like shapes can be labeled by two gold particles although the HIV-1 core is composed of ∼1,500 CA monomers (12, 58). This can be explained by specific features of EM immunolabeling that are dependent on the specificity of the antibody, accessibility, and number of antigens on thin sections, among other factors (59). Intranuclear CA complexes were detected by more gold particles than the cytoplasmic viral structures, which we believe is due to rearrangement of the viral CA (Fig. 2B, frames iv and v, and Fig. 2C, D, and E). It should be noted that, so far, the presence of multiple CA proteins inside the nucleus of infected mitotic cells has never been reported. To confirm the detection of intranuclear complexes, tomograms performed at 6 h postinfection revealed the formation of pearl necklace-like shapes composed of multiple CA proteins located in the nucleus (Fig. 2E). Often these nuclear CA structures are associated with viral IN (Fig. 3A). Importantly, similar complexes composed of multiple CA and IN proteins have been identified in HeLa cells and primary lymphocytes, revealing the organization of the viral CA proteins also in the main target cells of HIV-1 (Fig. 3C). Our results, thanks to the ability to combine the specific labeling of the retrotranscribed DNA (HIV-1 ANCHOR) with immunogold CA labeling, show that viral complexes observed during nuclear translocation could represent PICs. Thus far, the viral DNA has never been visualized associated with multiple CA proteins in dividing infected cells. Importantly, we observed that HIV-1 ANCHOR allowed the specific detection of the viral DNA, as shown by the use of drugs (Fig. 5). This is highlighted by using drugs that block different steps of early events of HIV-1 infection, such as nuclear import (PF74 drug) or reverse transcription (nevirapine) (Fig. 5). Bright spots identifying viral DNA were prevalently detected in the nuclei of untreated cells, in agreement with results obtained by real-time PCR that amplified two-long-terminal-repeat (2LTR) circles (Fig. 5B to D), commonly used to detect viral nuclear import (60). Thus, CLEM enabled visualization of the organization of HIV-1 PIC during nuclear translocation in HIV-1-permissive cells. It should be noted that HIV-1 ANCHOR can specifically label all nuclear viral DNA forms; thus, further experiments are needed to distinguish integration-competent viral complexes from dead end products. Independently of the fate of the PIC, HIV-1 ANCHOR technology combined with EM offered the opportunity to observe how viral CA proteins are reshaped near the NPC to lead the viral genome into the nucleus (Fig. 6A and B). OR-GFP can bind the viral DNA only if it is exposed, as indicated by our CLEM results, which support our model of viral core remodeling. In fact, complexes of multiple CA proteins associated with the retrotranscribed DNA can be visualized by CLEM before and during nuclear translocation (Fig. 6A and B). It is likely that the observed complexes contain complete retrotranscribed genomes. In agreement with this observation, previous studies showed that OR-GFP binds only double-stranded DNA (dsDNA) (61), meaning that the vDNA shrouded in the multiple-CA protein complex is a dsDNA. The ANCH3 sequence has been cloned in the *nef* gene, which means that the vDNA identified by CLEM might be a late reverse transcript since OR-GFP can only bind it after the completion of reverse transcription on both strands containing the ANCH3 sequence. Thus, all viral complexes carrying an accessible vDNA can be observed by fluorescence microscopy. As opposed to fluorescence microscopy, the visualization of viral complexes by EM coupled with gold labeling is complicated but has the advantage of yielding high-resolution images. Sections are not permeabilized, so only CA epitopes exposed on the surface of the section can be labeled by the anti-CA antibody and then by a secondary gold-labeled antibody. Indeed, a limited number of available epitopes can be recognized by the primary antibody in sections. The aforementioned reasons, together with the thorough sample fixation required for the EM, strictly limit the number of epitopes accessible to the antibody; therefore, we observed more vDNA signal than CA protein signal. Apart from such technical limitations, we do not expect to have all vDNA complexes colabeled with CA as a result of the asynchronous infection conditions used and the consequent presence of some CA-free nuclear vDNA, as previously shown by Chin et al. (30). On the other hand, most of the HIV-1 particles are known to undergo an abortive uncoating in the cytoplasm. Thus, we focused on those viral CA complexes that also contain vDNA; interestingly, these viral complexes were found in the vicinity of the NE. Overall, these results indicate that the viral complexes visualized by CLEM could potentially be PICs.

In summary, the viral CA is reshaped before crossing the NPC, decorating the viral DNA, and leading it into the nucleus. Similar CA-viral DNA complexes can be also found near the NE (Fig. 7A) and inside the host nuclei (Fig. 7B).

The presence of consistent shapes formed by multiple CA proteins in the nucleus (Fig. 2B, frames iv and v, Fig. 2C, D, and E, and Fig. 3A and C), as well as their association to the retrotranscribed DNA (Fig. 6A and B and Fig. 7A and B) would indicate that these CA forms are imported with the viral genome into the host nucleus. These results would support the evolution of the concept of viral uncoating, no longer seen as a complete loss of viral CA but as CA remodeling during nuclear import. Another novel aspect of our work is the development of the HIV-1 ANCHOR system, the first fluorescence DNA labeling technique shown to be compatible with TEM. This innovative technology allows us to follow the HIV-1 DNA shrouded by multiple CA proteins during and after nuclear entry in mitotic cells. In addition, we were able to live-track the viral DNA in the nucleus of infected cells following translocation through the pore (see Movies S1, S2, S3, and S4 in the supplemental material).

Of note, our study gives new information on the early steps of HIV-1 infection in dividing cells. It is known that mitotic cells with integrated viruses may persist for many years and undergo clonal expansion (62). Clonal expansion seems to be the major mechanism to generate an HIV-1 reservoir (63), considered to be established early during primary HIV-1 infection (64).

Overall, our results provide new notions about not only the organization of viral PICs but also the dynamics and the fate of the viral DNA inside the nucleus. Our data elucidate how the viral CA is remodeled to lead HIV-1 DNA into the nucleus of infected cells.

These findings and technology can be useful for future studies on other pathogens or to investigate the interplay of HIV-1 DNA with nuclear factors and chromatin environments.

## MATERIALS AND METHODS

### Cells.

HeLa P4R5 cells, a HeLa-CD4/LTR-*lacZ* indicator cell line expressing both CXCR4 and CCR5, were employed to assess viral infectivity (65) using a beta-galactosidase assay. 293T cells (ATCC) are human embryonic kidney cells used to produce lentiviral vectors and HIV-1 viruses; HeLa cells (ATCC) are derived from cervical cancer cells. CD4^+^ T cells were isolated from healthy donors and obtained via the Etablissement Français du Sang (EFS), Paris, France. Briefly, primary CD4^+^ T cells were purified from human peripheral blood by density gradient centrifugation (lymphocyte separation medium; PAA), followed by positive immunomagnetic selection with CD4 microbeads (Miltenyi). A day later, cells were activated by a T cell activation kit (Miltenyi) for 2 to 3 days at 37°C with interleukin-2 (IL-2)-containing medium (50 IU/ml); then cells were challenged with HIV-1 carrying the CA wild type or mutant. The percentage of p24-positive cells was obtained by cytofluorimetry acquisition and FlowJo analysis.

### Antibodies.

Antibodies used were the following: anti-actin conjugated to horseradish peroxidase (HRP) (sc-2357; Santa Cruz) (diluted 1:5,000), anti-p24 NIH183-H12-5C or AG3.0 (NIH AIDS Reagent Program) (for immunofluorescence [IF], diluted 1:400; for TEM, diluted 1:50), anti-HA high-affinity Ab (11867423001; Roche) (for TEM, diluted 1:50; for IF, diluted 1:500), goat anti-mouse Alexa Fluor Plus 488 and goat anti-rat Alexa 647 (A32723 and A21247, respectively; ThermoFisher Scientific); goat anti-mouse coupled to 10-nm gold particles(ab39619; Abcam), goat anti-rat coupled to 6-nm gold particles (ab105300; Abcam) (diluted 1:50), goat anti-rabbit coupled to 5-nm gold particles(ab27235; Abcam), anti-GFP rabbit (ab183734; Abcam) (for CLEM, diluted, 1:50), anti-GFP (632592; Clontech)(for Western blotting [WB], diluted 1:1,000), beta-actin conjugated to HRP (8226; Abcam) (for WB, diluted 1:2,500), goat anti-rabbit Alexa 488 (A11078; ThermoFisher Scientific) (for CLEM, diluted 1:50), anti-Nup1539 (kind gift from B. Burke; diluted 1:200), protein A-conjugated 10-nm gold particles (diluted 1:50; University Medical Center UMC, Utrecht, Netherlands).

### Time‐lapse microscopy.

HeLa P4R5 cells stably transduced with LV OR-GFP were plated in Hi‐Q4 microdishes (10,000 cells per chamber) (Ibidi). The following day, cells were infected with HIV-1 ΔEnv IN_HA_ ΔNef ANCH3/VSV-G. Transmission and fluorescence images were taken every 5 or 10 min for up to 96 h using a Nikon Biostation IMQ (40× objective) with 6 to 8 fields captured simultaneously for each condition or for up to 24 h by using a spinning-disk UltraView VOX (Perkin-Elmer) (63× objective), with one field of view for each experiment in 2D or 3D. Images were analyzed in Fiji or Imaris.

### WB and confocal immunofluorescence microscopy.

The correct expression of OR-GFP after LV transduction was evaluated in HeLa cells by Western blotting (WB). Proteins were extracted on ice from wild-type and LV OR-GFP transduced HeLa cells using radioimmunoprecipitation assay (RIPA) buffer (20 mM HEPES, pH 7.6, 150 mM NaCl, 1% sodium deoxycholate, 1% Nonidet P-40, 0.1% SDS, 2 mM EDTA, complete protease inhibitor [Roche Diagnostics]), and protein concentration was quantified using a detergent-compatible (DC) protein assay (Bio-Rad Laboratories) with bovine serum albumin (BSA) as a standard. Ten micrograms of total protein lysate was loaded onto SDS-PAGE 4 to 12% Bis-Tris gels (Invitrogen). Visualization was carried out using an ECL Plus Western blotting kit (GE Healthcare). Primary antibody used for WB was anti-GFP (632592; Clontech) (dilution, 1:1,000). Secondary conjugated antibodies used for Western blotting were beta-actin HRP-conjugated antibody (8226, diluted 1:2,500; Abcam) and anti-rabbit IgG HRP (sc2357; Santa Cruz). For immunofluorescence microscopy, HeLa P4R5 cells stably expressing OR-GFP or not were plated onto 12-mm diameter coverslips in 24-well plates the day before and then infected with HIV-1 ΔEnv IN_HA_ ΔNef ANCH3/VSV-G or HIV-1 ΔEnv IN_HA_/VSV-G at different MOIs and different times postinfection. The cells were then washed, fixed with 4% paraformaldehyde (PFA), permeabilized with 0.5% Triton X‐100 for 30 min, and blocked with 0.3% bovine serum albumin (BSA). All incubations were carried out at room temperature and were followed by ﬁve washes with phosphate-buffered saline (PBS). Cells were incubated with primary antibodies for 1 h and with secondary antibodies for 30 min. Antibodies were diluted in 0.3% BSA. Nuclei were stained with Hoechst (diluted 1:10,000; Invitrogen). Finally, cells were mounted onto glass slides (Thermo Scientiﬁc) with Prolong Diamond (Life Technologies). Confocal microscopy was carried out on a Zeiss LSM700 using a 63× objective. Representative medial sections or combined z‐stacks are shown as indicated in Fig. 1 and 5. Images were analyzed in Fiji.

### Viral infection and sample preparation for electron microscopy.

Eight million HeLa P4R5 or HeLa P4R5 OR-GFP transduced cells were seeded in a T75 flask and infected with 4,000 ng of p24 of either HIV-1 IN_HA_ or HIV-1 ANCH3 and incubated for 6 h. When a WT virus has been used to infect HeLa P4R5 cells or primary CD4^+^ T cells, an ultracentrifuged virus has been used in some cases in the presence of semen-derived enhancer of virus infection (SEVI) according to a published protocol (66, 67) (SEVI fibrils were kindly provided by Franck Kirchhoff). Infectivity was analyzed by beta-galactosidase assay or by fluorescence-activated cell sorting (FACS). Samples were prepared for EM as follows: cells were fixed by adding directly an equal volume of 8% paraformaldehyde and 0.2% glutaraldehyde in PHEM buffer (60 mM PIPES [piperazine-*N*,*N*′-bis(2-ethanesulfonic acid)], 25 mM HEPES, 2 mM MgCl_2_, 10 mM EGTA, pH 7.3) solution to the cell medium and incubated for 30 min. Next, the solution was exchanged by 4% paraformaldehyde diluted in PHEM buffer and incubated for 2 h at room temperature. Cells were further prepared for cryo-microtome sectioning and immunolabeled as described previously (68). Electron microscopy chemicals were purchased from Electron Microscopy Sciences (Hatfield, PA). For the CLEM experiments, before being stained with uranyl acetate for contrast, the samples were stained with 1 μM Hoechst for 20 min in water, washed, and incubated with a solution of 0.2 μm TetraSpeck fluorescent beads (ThermoFisher Scientific) diluted 1:50 in PHEM buffer, pH 7.3, for 20 min and washed four times for 2 min with water. The samples were mounted in a glass-bottom petri dish (Miltenyi Biotec) with a drop of SlowFade Diamond antifade mountant (ThermoFisher Scientific). The imaging process gave a mosaic map of the sections in the blue, green, and far-red channels using a 63× (1.4 numerical aperture [NA]) objective with a Leica DSM6000 microscope equipped with Orca Flash 4.0 LT camera (Hamamatsu Photonics). Then the grids were recovered by pouring 10 μl of water underneath them. Grids were washed, contrasted, and prepared for TEM as specified above. For the cryo-EM observation, the samples were prepared as described above. After immunolabeling the grids were embedded with a 1:1 mixture of 2% methylcellulose and 2.3 M sucrose and then vitrified by plunge freezing with an EMGP plunge freezer (Leica) at 30°C and 90% humidity.

### Electron microscopy data collection and image processing.

Sections, at room temperature or under cryo-conditions, were transferred and imaged in a Tecnai T12 transmission EM operating at 120 or 80 kV and equipped with a Gatan Ultrascan 4000 camera. Multiscale mapping and tilt series acquisitions in areas of interest were processed by Serial EM software (69). In the case of cryo-samples, low-dose conditions and bi-directional tilt schemes were used during acquisition. Tilt series stacks were initially aligned using cross-correlation, and the alignments were further refined using immunogold beads as registration fiducials in IMOD (70). Tomograms were reconstructed with the weighted back-projection method and filtered to assist manual segmentation with IMOD.

The correlation between fluorescence and electron microscopy images was achieved using the following protocol: (i) z-stacks of every frame of the mosaic were projected with the Fiji plug-in extended depth of field (71); (ii) the frames were aligned and blended to generate a fluorescence map of the complete section using Mosaic J (72); (iii) the same cells were identified in both fluorescence and low-resolution TEM section maps; (iv) high-precision correlation was obtained by identifying TetraSpeck positions in high-resolution fluorescence and TEM images using the ecCLEM plug-in (57) of Icy (56).

### Quantitative PCR.

Total cellular DNA was isolated using a QIAamp DNA microkit (Qiagen) at 7 and 24 h postinfection. The genomic DNA was treated for 1 h at 37°C with DpnI. Nevirapine (10 mM) was used in infected cells as a control of the experiment. Late reverse transcription products at 7 h postinfection were measured by real-time PCR using previously described primers and probe (60); 2LTR-containing circles were detected using primers MH535/536 and probe MH603, using for a standard curve the pUC2LTR plasmid, which contains the HIV-1 2LTR junction. Integration was assessed by Alu-PCR, using primers designed in the U3 region of LTR (4), which is deleted in the LVs carrying OR-GFP but not in the LTR of HIV-1 used to challenge cells stably expressing OR-GFP and control cells. The standard curve was prepared as follows: DNA generated from infected cells was endpoint diluted in DNA prepared from uninfected cells, and serial dilutions were made starting from 50,000 infected cells. Each sample amplified contained 10,000 infected cells mixed with 40,000 uninfected cells. The control of the first-round PCR was the amplification without Alu primers but only U3 primers (4). Dilutions of 1:10 of the first round were amplified by real-time PCR (4). Internal controls were included, such as infection in the presence of raltegravir (RAL) (10 μM). LRT, 2LTR, and Alu-PCRs were normalized by amplification of the housekeeping gene actin (4).

### Plasmids and viral production.

The HIV-1 ΔEnv IN_HA_ ΔNef ANCH3 plasmid was obtained by PCR using as the template the plasmid pANCH3 (NeoVirTech [NVT]) (53). Primers containing the restriction site for XhoI were applied to amplify ANCH3 sequence (∼1 kb). The amplicon was cloned in the XhoI site of HIV-1 ΔEnv IN_HA_. The final plasmid, HIV-1 ΔEnv IN_HA_ ΔNef ANCH3 has been sequenced. The LV cytomegalovirus (CMV) OR-GFP was generated by cloning by PCR the OR-GFP from the plasmid pOR-GFP (NeoVirTech) (53) in pTripCMVGFP. We amplified OR-GFP (∼1.8 kb) using specific primers to amplify the cDNA OR-GFP (NeoVirTech property); the primers, named OR-GFP 5′ and OR-GFP 3′, contain restriction sites for AgeI and SgrDI to allow cloning in pTripCMVGFP. The resulting plasmids were accurately sequenced and compared with ANCH3 and OR-GFP sequences from respective plasmids obtained by NeoVirTech. The ANCHOR technology is the exclusive property of NVT. Lentiviral vectors and HIV-1 viruses were produced by transient transfection of 293T cells using calcium phosphate coprecipitation. Lentiviral vectors were produced by cotransfection of 10 μg of the transfer vector LV OR-GFP with 2.5 μg of pMD2 VSV-G and 10 μg of ΔR8.74 plasmids. HIV-1 viruses were produced by cotransfection with calcium phosphate with HIV-1 LAI (BRU) ΔEnv virus (NIH), or with the modified versions HIV-1 ΔEnv IN_HA_ (41) (kindly provided by Fabrizio Mammano) or the HIV-1 ΔEnv IN_HA_ ΔNef ANCH3 and VSV-G envelope expression plasmid pHCMV-G (VSV-G). The viruses collected from 293T cells at 48 h posttransfection were ultracentrifuged at 4°C for 1 h at 22,000 rpm. Virus normalizations were performed by p24 enzyme-linked immunosorbent (ELISA) according to the manufacturer’s instructions (Perkin Elmer) and by real-time PCR. The titer of each virus was calculated by qPCR as the number of transducing units (TU) per milliliter and then used to calculate the MOI (number of TU/number of cells). Infectivity has been tested by beta-galactosidase assay (Merck), with activity measured at 48 h postinfection according to the manufacturer’s instructions, using a microplate fluorimeter (Victor, Perkin Elmer). Protein quantification by a Bio-Rad protein assay was carried out on the same lysates to normalize the beta-galactosidase data for protein content.

## Supplementary Material

Supplemental file 1

Supplemental file 2

Supplemental file 3

Supplemental file 4

Supplemental file 5

Supplemental file 6

Supplemental file 7

Supplemental file 8

Supplemental file 9

Supplemental file 10
