# Supplementary material for: Remodeling of the Core Leads HIV-1 Preintegration Complex into the Nucleus of Human Lymphocytes
Source: J Virol. 2020 May 18;94(11):e00135-20. doi: 10.1128/JVI.00135-20 (PMC7269431; doi:10.1128/JVI.00135-20)
Supplement: Supplemental file 10 [file JVI.00135-20-s0010.pdf]

JVI00135-20 Supplemental Movie Legends

Movie 1(A). HeLa P4R5 cells stably expressing OR-GFP infected with HIV-1ΔEnvINHAΔNef ANCH3/VSVG and imaged at the biostation for 71h p.i.

Movie 1(B). Uninfected HeLa P4R5 cells (control) stably expressing OR-GFP imaged for 71h at the biostation.

Movie 2(A). 3D reconstruction by Imaris software of HeLa P4R5 cells stably expressing OR-GFP infected with HIV-1ΔEnvINHAΔNef ANCH3/VSV-G and imaged by spinning disk.

Movie 2(B). 3D live imaging of HeLa P4R5 cells stably expressing OR-GFP infected with HIV-1ΔEnvINHAΔNef ANCH3/VSV-G 7h post-infection.

Movie 2(C). 2D live imaging of HeLa P4R5 cells stably expressing OR-GFP infected with HIV-1ΔEnvINHAΔNef ANCH3/VSV-G from 4h post-infection.

Movie 3(A). HeLa P4R5 cells stably expressing OR-GFP infected with HIV-1ΔEnvINHAΔNef ANCH3/VSVG and imaged at the biostation for 26h p.i. in absence of PF74.

Movie 3(B). HeLa P4R5 cells stably expressing OR-GFP infected with HIV-1ΔEnvINHAΔNef ANCH3/VSVG and imaged at the biostation for 26h p.i. in presence of low dose of PF74

Movie 3(C). HeLa P4R5 cells stably expressing OR-GFP infected with HIV-1ΔEnvINHAΔNef ANCH3/VSVG and imaged at the biostation for 26h p.i. in presence of high dose of PF74 drug.

Movie 4(A). HeLa P4R5 cells stably expressing OR-GFP infected with HIV-1ΔEnvINHAΔNef ANCH3/VSVG and 3D imaged by confocal at 24h post infection.

Movie 4(B). HeLa P4R5 cells stably expressing OR-GFP infected with HIV-1ΔEnvINHAΔNef ANCH3/VSVG in presence of 10µM of nevirapine and 3D imaged by confocal at 24h post infection.
